# Determining Zygosity With Multiplex Kompetitive Allele‐Specific PCR (mxKASP) Genotyping

**DOI:** 10.1002/ece3.71642

**Published:** 2025-06-21

**Authors:** Willem R. M. Meilink, Manon C. de Visser, Anagnostis Theodoropoulos, Michael Fahrbach, Ben Wielstra

**Affiliations:** ^1^ Institute of Biology Leiden Leiden University Leiden the Netherlands; ^2^ Naturalis Biodiversity Center Leiden the Netherlands; ^3^ Blijdorp Conservation & Science Center Royal Rotterdam Zoological & Botanical Gardens Rotterdam the Netherlands; ^4^ Niedernhaller Str. 8/2 Criesbach Germany

**Keywords:** balanced lethal system, multiplex PCR, sex chromosome, supergene, *Triturus*

## Abstract

We introduce multiplex Kompetitive Allele‐Specific PCR (mxKASP): a modification of “classical” KASP genotyping that allows zygosity to be determined in diploid organisms. Rather than targeting a SNP associated with a single marker, mxKASP targets two non‐homologous markers. We show proof of concept by applying mxKASP to the balanced lethal system in *Triturus* newts, in which individuals are known to possess either: (1) zero copies of the 1A version of chromosome 1 and two copies of the 1B version; (2) one copy of 1A and one copy of 1B; or (3) two copies of 1A and zero copies of 1B. mxKASP is successful in amplifying both a 1A and a 1B marker in a single reaction (if present), allowing the zygosity of individuals to be inferred. We independently confirm our mxKASP results with a multiplex PCR approach. We argue that mxKASP can be applied to rapidly and economically determine zygostity in diploid organisms, for a large number of samples at once.

## Graphical abstract

Our study introduces mxKASP, a modified Kompetitive Allele‐Specific PCR technique that allows for determination of zygosity in diploid organisms by targeting two entirely different, non‐homologous markers. This is different from traditional KASP, in which a single marker is targeted, but with two different, SNP‐specific primers.

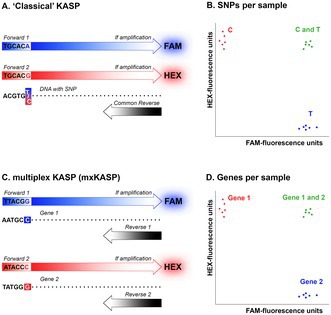

## 1 Introduction

In diploid organisms, a stretch of DNA, such as a gene or even an entire chromosome, is *hemizygous* if only a single copy is present in the genome. The enormous impact (hemi)zygosity can have on the phenotype is perhaps best showcased by *sex chromosomes*. In species with genetic sex determination, the heterogametic sex is hemizygous for the region that underpins sex, whereas the homogametic sex is homozygous (Bachtrog et al. 2014). Zygosity also varies in the case of *supergenes*, which are physically linked genes that are inherited together because recombination is suppressed (Thompson and Jiggins 2014; Berdan, Flatt, et al. 2022; Schwander et al. 2014). In fact, sex chromosomes are generally considered a kind of supergene (Schwander et al. 2014). Supergenes come in (at least) two versions, and which version(s) an individual possesses—two different versions or the same version twice—may greatly influence its phenotype. Hence, zygosity is of great biological relevance, and techniques that allow zygosity to be determined would be widely applicable, given the widespread occurrence of sex chromosomes and other supergenes across the tree of life. Ideally, such a technique would be highly capable of processing many samples simultaneously and rapidly at low cost.

**FIGURE 1:**
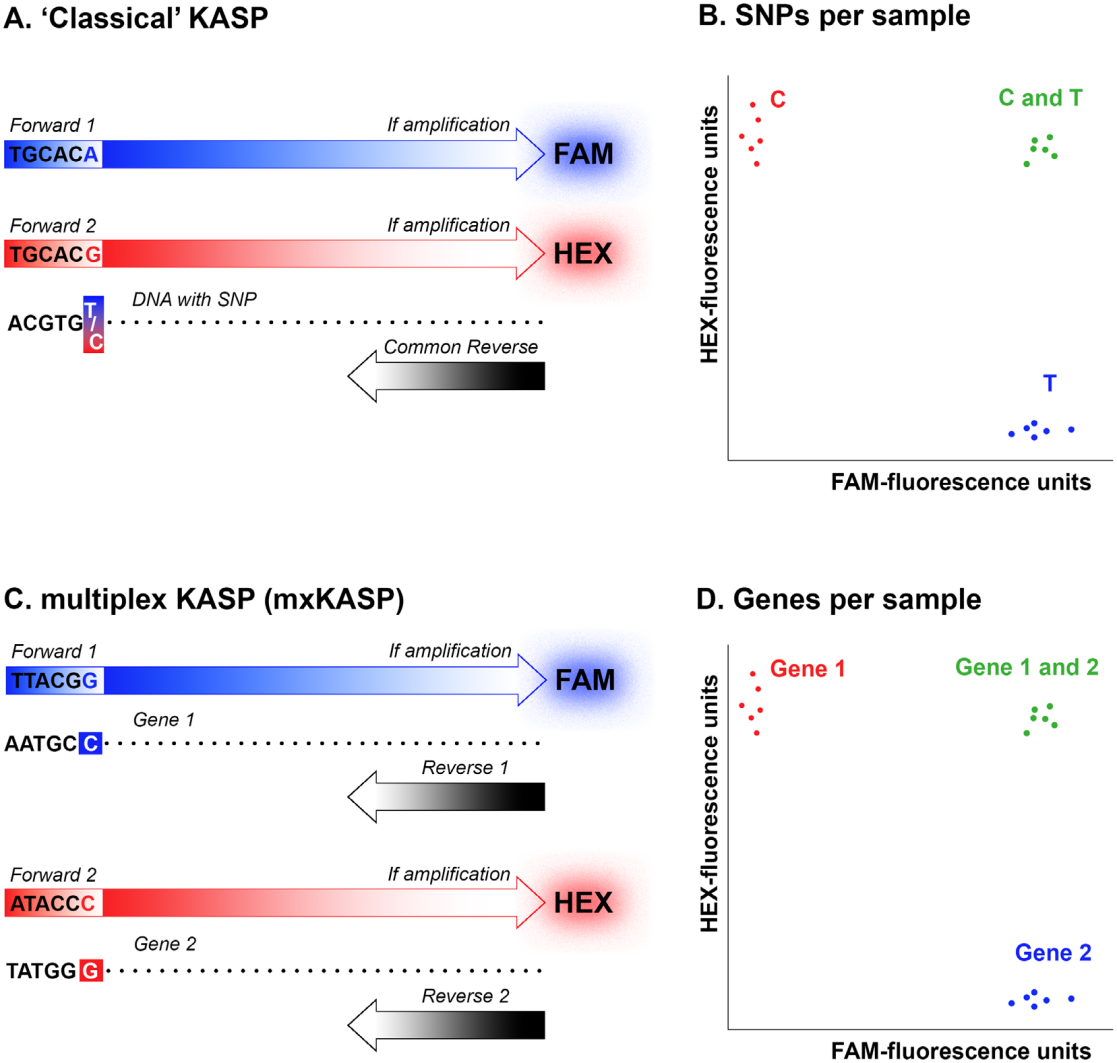
Schematic showing the different approach between KASP and mxKASP. (A) In KASP two forward primers differ in their last nucleotide as defined by the targeted SNP. Depending on which SNP variant is present, amplification leads to a fluorescent signal of FAM, HEX, or both. (B) For individuals homozygous for one SNP variant (in this case C) only the HEX signal is amplified (red dots), while for individuals homozygous for the other SNP variant (T) only the FAM signal is amplified (blue dots); for heterozygous individuals (C/T) both fluorescent signals are emitted (green dots). (C) In mxKASP two pairs of forward and reverse primers are designed for different (non‐homologus) genes. (D) The presence of only one or the other gene will result in either the HEX (in this case for gene 1, red dots) or the FAM (gene 2, blue dots) signal to be amplified, while if both genes are present, both fluorescent signals are emitted (green dots).

Kompetitive Allele Specific PCR (KASP) is an efficient and cost‐effective method for genotyping SNPs in a large number of samples (Semagn et al. 2013). In this method, typically a single common reverse primer is used in combination with two allele‐specific forward primers that differ in the last nucleotide, defined by the targeted SNP. These primers also possess a tail that is complementary to one of the two quenched fluor‐labeled oligos; one with FAM, one with HEX. When thermal cycling exponentially increases one tail (in individuals homozygous for one or the other SNP) or both tails (in heterozygous individuals), the complementary label is no longer quenched, resulting in a corresponding fluorescent signal. However, with a slight modification, KASP could, in theory, also be used to efficiently genotype markers for zygosity. Rather than targeting a single marker with SNP‐specific primers, as in “classical” KASP, two different (i.e., non‐homologous) markers that are positioned on the genomic region relevant for zygosity could be targeted, to determine the presence or absence of these markers. To this aim we use two distinct primer pairs, each with a different quenched fluor‐labeled oligo, in a single reaction (Figure 1). We call this modification multiplex KASP (mxKASP).

We show proof of concept by applying mxKASP in a balanced lethal system. In a balanced lethal system two versions of a chromosome are involved (Berdan, Blanckaert, et al. 2022; Wielstra 2020; Muller 1918). Healthy individuals possess a single copy of both chromosome versions, meaning they are reciprocally hemizygous, while diseased individuals possess two identical chromosome versions, meaning they are homozygous for one or the other version. The best studied example of a balanced lethal system concerns the *chromosome 1 syndrome* in newts of the genus *Triturus*, in which two versions of chromosome 1 are involved: 1A and 1B (Sessions et al. 1988; Sims et al. 1984; Macgregor and Horner 1980). Only individuals that possess both versions of chromosome 1 (1A1B) survive embryonic development, whereas individuals that have the same version twice (1A1A or 1B1B), and therefore lack the alternate version, perish inside the egg. We design KASP markers that are positioned either on 1A or 1B, with the expectation that only one or the other marker would be amplified in diseased (1A1A or 1B1B) individuals, whereas both should amplify in healthy (1A1B) individuals.

## 2 Material and Methods

### 2.1 Sample Selection

Embryos representing nine *Triturus* species (Wielstra and Arntzen 2016), including the two distinct Balkan and Italian clades of *T. carnifex* (Wielstra et al. 2021) were included in the study. We included three individuals per genotype (1A1B, 1A1A, and 1B1B), adding up to 90 *Triturus* individuals in total. We also included four individuals of *Lissotriton vulgaris* , a species that is closely related to *Triturus* (Rancilhac et al. 2021), but that is not affected by the balanced lethal system (Horner and Macgregor 1985).

### 2.2 Marker Selection

De Visser, France, Paulouskaya, et al. (2024) produced DNA sequence data for c. 7 k nuclear DNA markers from across the genome of three healthy (1A1B) and six diseased (three 1A1A and three 1B1B) individuals for the same 10 *Triturus* clades as above (i.e., 90 samples in total). This dataset includes dozens of 1A‐linked markers (not present in 1B1B individuals) and 1B‐linked markers (not present in 1A1A individuals), next to thousands of additional markers positioned elsewhere on the genome. We also included three individuals of *Lissotriton vulgaris* from De Visser, France, McCartney‐Melstad, et al. (2024). We took sequences of the (in *Triturus*) 1A‐linked marker *PLEKHM1*, the 1B‐linked marker *NAGLU* and the “control marker” *CDK17*, found on another linkage group than the 1A and 1B marker (France et al. 2024; De Visser, Van Der, Cvijanović, et al. 2024). Sequences were aligned and trimmed in MEGA 11 (Tamura et al. 2021), using the Muscle alignment tool set. Next, Unipro UGENE v.33 (Okonechnikov et al. 2012) was used to generate consensus sequences, with polymorphisms encoded by the relevant IUPAC codes. The consensus sequences were generated using an 80% threshold value.

### 2.3 mxKASP

Four primer pairs were designed for the 1A‐linked marker *PLEKHM1* and an additional four for the 1B‐linked marker *NAGLU*, using LGC genomics' Kraken v.23.11. This resulted in 16 (four by four) possible primer pair combinations (Table S1). KASP assays were prepared in a total volume of 200 μL, with 24 μL of both forward primers (10 μM), 60 μL of both reverse primers (10 μM), and 32 μL purified water. Next, KASP mix was made for each primer combination and contained 1.26 μL KASP assay, 102.8 μL KASP master mix, and 102.8 μL purified water. All 16 combinations were tested in a 1536 well plate with 1 μL of KASP mix and 1 μL of DNA for 93 samples (90 *Triturus*, 3 *Lissotriton*), as well as a negative control (no DNA). mxKASP was performed in a hydrocycler, beginning with 10 cycles with an annealing temperature decreasing from 61°C to 55°C, followed by 26 cycles at 55°C. Two additional three‐cycle recyclings were performed with annealing at 57°C. Fluorescence was measured using a PHERAstar plate reader, and data were analyzed using Kraken.

Two primer pair combinations (1A‐linked primer pairs NAGLU.2 or NAGLU.3 with 1B‐linked marker primer pair PLEKHM1.3; Table 1) showed separation into three clear groups (see results), corresponding to the three potential genotypes (1A1A, 1A1B, 1B1B). These two combinations were repeated in a 384‐well plate to confirm the results. The KASP mix for each primer pair consisted of 7.2 μL assay, 194.4 μL KASP master mix, and 194.4 μL purified water. Here, 3 μL of DNA from the same samples, next to a negative control, were combined with 3 μL of KASP mix, with identical downstream procedures as before.

**TABLE 1:** List of primers used for mxKASP (top six) and mxPCR (bottom six).

Chromosome	Marker	Primer	Sequence	Dye
1A	PLEKHM1	PLEKHM1.3_F	AGTTCTCAAAGCAGGTCCGGTC^a^	FAM
1A	PLEKHM1	PLEKHM1.3_R	GAGTTGTCTCCATTTGAGTTACGCC	NA
1B	NAGLU	NAGLU.2_F	AGCTTGGTTGAACTCATCCTGGT^b^	HEX
1B	NAGLU	NAGLU.3_F	GCTAGTGGCTAAGGATTGAGCAT^b^	HEX
1B	NAGLU	NAGLU.2_R	ACRCTAGTGGAATGCCTCAACACG	NA
1B	NAGLU	NAGLU.3_R	CACTTCCTACTTGGCAGTTGGCTTT	NA
Chromosome	Marker	Primer	Sequence	Product length (bp)
1A	PLEKHM1	PLEKHM1_Fm	CCAGTGGCATTTTCAGGTCC	336
1A	PLEKHM1	PLEKHM1_R3	GGGAGTTCTTCTGCGAGTTG	
1B	NAGLU	NAGLU_F3	TGGTTGAACTCATCCTGGTG	416
1B	NAGLU	NAGLU_R3	GTTGATGTCACAAGGCAAGC	
Control	CDK17	CDK17_F1	GGCATGGGAAGAACAGAAGA	537
Control	CDK17	CDK17_R1	CCATCTGCTTGGACTGTTGA	

^a^ The location of the LGC‐patented dye‐complementary tail sequence: binds to FAM.

^b^ The location of the LGC‐patented dye‐complementary tail sequence: binds to HEX.

### 2.4 mxPCR

To confirm our mxKASP results we designed a multiplex PCR (mxPCR) protocol. Primers were developed in Primer3Plus (Untergasser et al. 2012), aiming for a size difference (to be able to distinguish markers by gel electrophoresis) between the 1A‐linked marker *PLEKHM1* (200–300 bp), the 1B‐linked marker *NAGLU* (350–450 bp) and the control marker *CDK17* (> 500 bp). *CDK17* is located on a linkage group that does not correspond to chromosome 1 (France et al. 2024). All other parameters were kept the same (Table 1). The mxPCR was performed using QIAgen multiplex PCR master mix. PCR was performed in 12 μL reactions, containing 0.06 μL of all forward and reverse primers (at 10 μM concentration, resulting in a 0.05 μM end concentration of each primer), 6 μL QIAGEN multiplex PCR master mix, 4.64 μL purified water, and 1 μL of DNA. PCR conditions were as follows: a hot start for 15 min at 95°C, followed by 35 cycles of denaturation for 30 s at 95°C, annealing for 1 min at 56°C, and extension for 1 min at 72°C, and a final 10 min extension at 72°C.

## 3 Results

**FIGURE 2:**
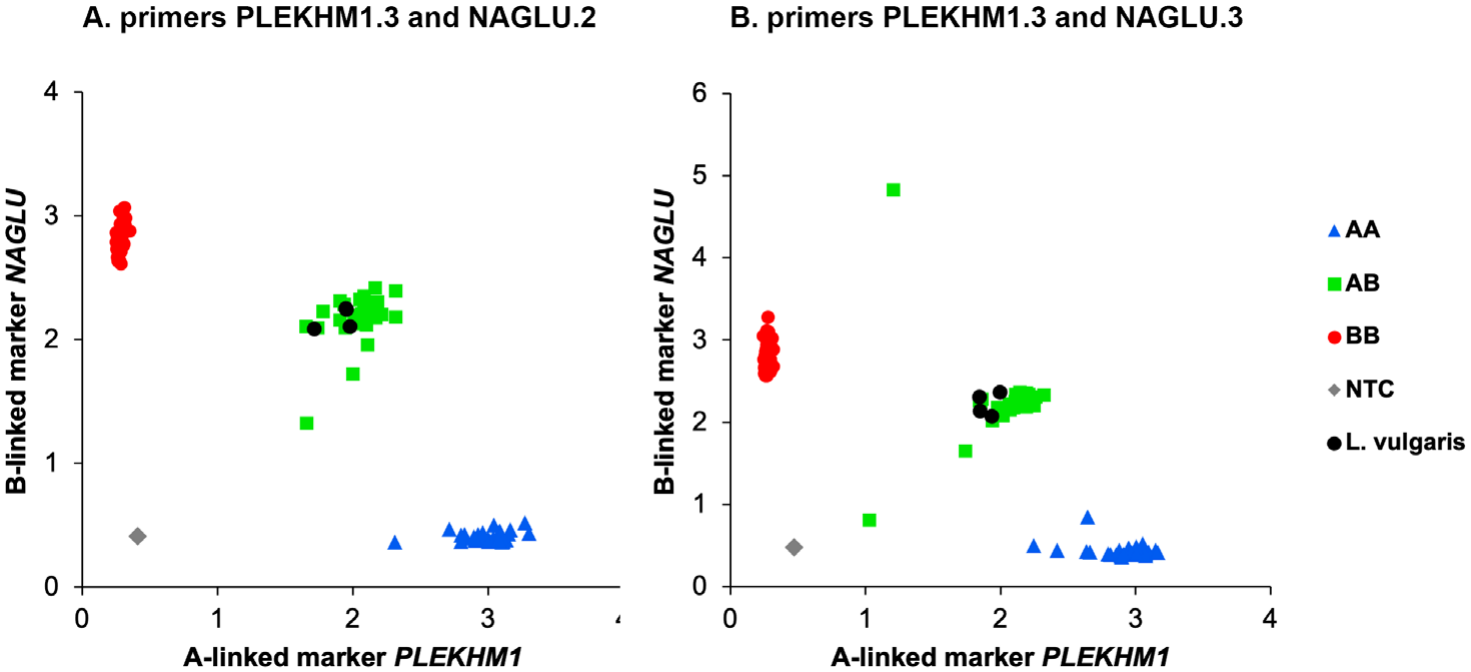
KASP results for two markers on different chromosomes, one on chromosome 1A (*PLEKHM1*) and one on chromosome 1B (*NAGLU*). Primers PLEKHM1.3 and NAGLU.2 (A. left panel) lead to complete separation of the samples into three clusters, while primers PLEKHM1.3 and NAGLU.3 (B. right panel) show a similar division with outliers for two 1A1B individuals. The *x*‐axis reflects FAM and the *y*‐axis HEX fluorescence units (*y*‐axes are not to scale).

In our mxKASP experiment, two out of 16 combinations of primer pairs resulted in a clear separation of three genotype clusters of the samples (Figure 2; Table S2). The combination PLEKHM1.3 and NAGLU.2 showed allocation of all individuals into the expected genotype clusters, while two 1A1B samples were outliers for the combination of primers PLEKHM1.3 and NAGLU.3 (Figure 2). The four *Lissotriton* samples amplified for both PLEKHM1.3 and NAGLU.2/3.

**FIGURE 3:**
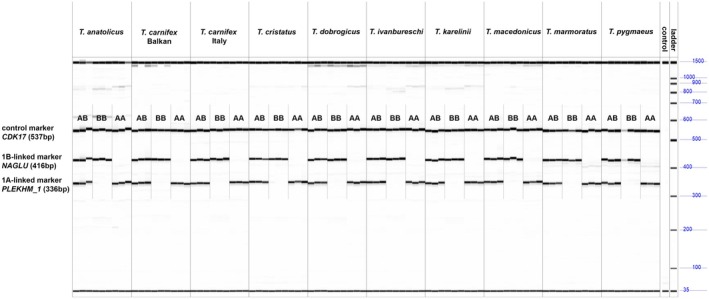
Multiplex PCR results obtained with a Fragment Analyzer. Three genotypes (healthy 1A1B or diseased 1A1A or 1B1B) can be distinguished for 10 *Triturus* (candidate) species.

The mxPCR amplification genotyped each individual for all 10 candidate *Triturus* species as expected (Figure 3). The control marker *CDK17* (top band) is visible for all individuals, whereas the 1B marker *NAGLU* (middle band) and the 1A marker *PLEKHM1* (lowest band) are only both visible in healthy (1A1B) individuals. The diseased individuals miss either the 1A marker (1B1B) or the 1B marker (1B1B). For the distantly related *Lissotriton*, the marker *PLEKHM1* did not amplify (results not shown).

## 4 Discussion

Our modification of KASP, mxKASP, proved to be successful in determining zygosity. Instead of targeting different SNPs on a single marker, as is typically done with KASP, we show that it is possible to target completely different, non‐homologous markers. We use mxKASP to independently establish the presence or absence of two markers, which allows us to infer zygosity in the *Triturus* balanced lethal system. “Classical” KASP has been used before to determine zygosity by targeting a SNP distinguishing the X and Y chromosomes, that could be identified because it co‐segregated with the male‐specific region (She et al. 2021). Our approach does not require such a SNP discovery step; rather it would directly target a marker private to the male‐specific region, next to a marker present in both males and females.

Earlier KASP modifications could potentially inform on zygosity. “Duplex KASP (dKASP)” was shown to be able to amplify two SNPs on two paralogous genes in a single run, with one of the two potential SNP variants for each paralog being present, resulting in a fluorescent signal (Jiang et al. 2023). This could be applicable to non‐homologous genes as well, but depends on the presence of SNPs, which is not the case for mxKASP. The KASP modification “4‐fluorescent KASP” amplifies two different SNPs, positioned on two non‐homologous markers, in a single run, by using four rather than the standard two fluorescent cassettes (Suo et al. 2020). Our mxKASP approach is more straightforward because it does not require additional fluorescent cassettes and is not dependent on the presence of SNPs on the markers of interest.

Over the last two decades, KASP has become increasingly popular as a uniplex SNP genotyping platform for the agricultural, biological, forensic, and medical sciences (Kalendar et al. 2022; Majeed et al. 2018; Kaur et al. 2020). There are several advantages of KASP, including modifications such as mxKASP, that make it attractive for broad application when considering many individuals for a few markers. (1) KASP is fast, simple, and scalable. Filling out DNA and chemicals is accomplished with robotics, and the PCR program takes under 2 h. There is no library preparation or sequencing involved. Data interpretation is practically automated and instantaneous, and the output is produced in a user‐friendly format. Failed calls can easily be repeated, and it is straightforward to add additional samples and markers at a later stage. (2) KASP can process a large number of samples. Reactions can be run in 96, 384, or 1536 well‐plates, with up to 14 plates per PCR program (Semagn et al. 2013). Therefore, c. 100.000 genotype calls can be produced in a day with a single KASP pipeline. (3) KASP is cheap. When not taking into account DNA extraction (which would be required for any genotyping method) or labor (which is relatively minimal for KASP compared to other methods), it has been estimated that the raw cost of a genotype call with KASP approximates €0.30 (Wielstra et al. 2016).

In comparison to KASP, the capacity of multiplex PCR (Henegariu et al. 1997) is limited by the number of available PCR machines, and data interpretation is relatively time‐consuming, relying on labor‐intensive gel or capillary electrophoresis. While zygosity can simply be inferred from the presence/absence of markers, methods such as quantitative PCR (Kralik and Ricchi 2017), digital droplet PCR (Hindson et al. 2013), digital PCR (Vogelstein and Kinzler 1999) and TaqMan (Shen et al. 2009) could also provide insights on relative copy numbers. The downside of these approaches is that they are more labor‐intensive and expensive and less geared towards high‐throughput compared to KASP.

Acquiring clean‐cut KASP results depends—as is the case for any genotyping method—on the quality of input DNA (Lear et al. 2018; Cavanaugh and Bathrick 2018; Brusa et al. 2021; Hsiao 2019). However, KASP only requires low quantity and quality DNA. Therefore, if KASP is combined with quick and cheap upstream methods, this opens up possibilities for scientists to upscale their research to the point of being able to sample, extract, and genotype thousands of individuals in a matter of days (Wielstra et al. 2016). For this reason, KASP has already been embraced as a more economical choice for quality control analysis in for instance plant breeding and seed systems, rather than genotyping by sequencing (Ertiro et al. 2015; Semagn et al. 2013). The same applies to the clinical and medical fields, which incorporate KASP to rapidly and accurately identify blood groups for blood donors (Krog et al. 2019), as well as to screen for genetic susceptibility to diseases such as Parkinson's and thrombosis (Altwayan et al. 2023; Landoulsi et al. 2017).

We foresee multiple applications in which our KASP modification, mxKASP, can be used for large‐scale zygosity level determination. The method can be used to improve animal welfare. For instance, mxKASP could replace capillary electrophoresis of PCR products or real‐time quantitative PCR previously recommended for use in the poultry industry to sex embryos *in ovo* to reduce male chick culling (He et al. 2019). Any PCR‐based method would avoid the controversial approach of genetically modifying layer hens for determining the sex of their eggs (Bruijnis et al. 2015). mxKASP could also prove valuable in a conservation context, for example, in species where sex is influenced by both a genetic and a thermal component (Hattori et al. 2012; Geffroy and Wedekind 2020). Circa 1/3 of fish species thought to exhibit only temperature‐dependent sex determination appear to possess genetic sex determination too, and which one predominates depends on environmental factors (Ospina‐Alvarez and Piferrer 2008). Global warming threatens such species by biasing sex ratios. As more sex‐linked markers are discovered, more genotype/phenotype mismatches will presumably be revealed (Edmands 2021; Geffroy and Wedekind 2020). Finally, mxKASP can be used to detect zygosity in fundamental eco‐evolutionary studies of supergene systems. For example, to test the ratios of different genotypes when a particular supergene version is assumed to carry recessive deleterious mutations (Kupper et al. 2016; Tuttle et al. 2016).

## Author Contributions

**Willem R. M. Meilink:** writing – original draft (equal). **Manon C. de Visser:** writing – original draft (equal). **Anagnostis Theodoropoulos:** writing – original draft (equal). **Michael Fahrbach:** writing – original draft (equal). **Ben Wielstra:** writing – original draft (equal).

## Conflicts of Interest

The authors declare no conflicts of interest.

## Supporting information


Data S1.

Tables S1–S2.

## Data Availability

All generated data has been published in this paper and can be found in the Supporting Information tables online.
